# Clinical Data, Chest Radiograph and Electrocardiography in the Screening for Left Ventricular Hypertrophy: The CAR_2_E_2_ Score

**DOI:** 10.3390/jcm11133585

**Published:** 2022-06-22

**Authors:** Patrycja S. Matusik, Amira Bryll, Agnieszka Pac, Tadeusz J. Popiela, Paweł T. Matusik

**Affiliations:** 1 Department of Diagnostic Imaging, University Hospital, 30-688 Kraków, Poland; patrycja.s.matusik@gmail.com; 2 Department of Radiology, Faculty of Medicine, Jagiellonian University Medical College, 30-688 Kraków, Poland; bryllamira@gmail.com (A.B.); msjpopie@cyf-kr.edu.pl (T.J.P.); 3 Chair of Epidemiology and Preventive Medicine, Faculty of Medicine, Jagiellonian University Medical College, 31-034 Kraków, Poland; agnieszka.pac@uj.edu.pl; 4 Department of Electrocardiology, Institute of Cardiology, Faculty of Medicine, Jagiellonian University Medical College, 31-202 Kraków, Poland; 5 Department of Electrocardiology, The John Paul II Hospital, 31-202 Kraków, Poland

**Keywords:** clinical data, chest X-ray, electrocardiogram, cardiac magnetic resonance imaging, left ventricular hypertrophy, screening, diagnostics

## Abstract

Left ventricular hypertrophy (LVH) is associated with adverse clinical outcomes and implicates clinical decision-making. The aim of our study was to assess the importance of different approaches in the screening for LVH. We included patients who underwent cardiac magnetic resonance (CMR) imaging and had available chest radiograph in medical documentation. Cardiothoracic ratio (CTR), transverse cardiac diameter (TCD), clinical and selected electrocardiographic (ECG)-LVH data, including the Peguero-Lo Presti criterion, were assessed. CMR–LVH was defined based on indexed left ventricular mass-to-body surface area. Receiver operating characteristics analyses showed that both the CTR and TCD (CTR: area under the curve: [AUC] = 0.857, *p* < 0.001; TCD: AUC = 0.788, *p* = 0.001) were predictors for CMR–LVH. However, analyses have shown that diagnoses made with TCD, but not CTR, were consistent with CMR–LVH. From the analyzed ECG–LVH criteria, the Peguero-Lo Presti criterion was the best predictor of LVH. The best sensitivity for screening for LVH was observed when the presence of heart failure, ≥40 years in age (each is assigned 1 point), increased TCD and positive Peguero-Lo Presti criterion (each is assigned 2 points) were combined (CAR_2_E_2_ score ≥ 3 points). CAR_2_E_2_ score may improve prediction of LVH compared to other approaches. Therefore, it may be useful in the screening for LVH in everyday clinical practice in patients with prevalent cardiovascular diseases.

## 1. Introduction

The demarcation between the normal and the pathological clinical findings is crucial. Clinical, chest radiograph and electrocardiographic (ECG) data might be helpful in this regard, especially in patients suspected of having or with cardiovascular diseases. Among others, it is important to be familiar with the abnormal appearance and dimensions of the cardiac silhouette on the chest radiograph (X-ray) before diagnosing cardiac enlargement [1]. Generally, cardiac enlargement on a chest radiograph is defined as a cardiothoracic ratio (CTR) of greater than 0.5 in posterior-anterior view [2,3]. Another indicator of cardiac enlargement is increased transverse cardiac diameter (TCD). Different TCD cut-off points have been described, while the values of 155 mm in men or 145 mm in women in posterior-anterior view are most commonly determined as increased [4]. However, there are limited data in the literature describing clinically significant abnormal chest radiograph features. It was demonstrated that CTR is sensitive and has a strong negative predictive value for screening for left ventricular (LV) enlargement by studies performed so far [2]. TCD is a more direct measure of cardiac size and a moderate correlation between TCD and LV end-diastolic volume (LVEDV) measured by cardiac magnetic resonance (CMR) was found [1]. However, the clinical value of CTR and TCD in prediction of LV hypertrophy (LVH) is poorly known [5].

Increased LV mass (LVM) may arise from multiple molecular mechanism, is associated with adverse clinical outcomes and may implicate clinical decision-making [6,7]. CMR imaging is considered the gold standard for assessment of LVM [8]. In comparison to chest radiograph, this imaging modality is not influenced by some extracardiac factors such as extensive thoracic fat deposits or chest wall expansion. However, CMR is not always available, is more complex in assessment and is relatively expensive. Those factors limit the use of CMR as a screening tool [9]. Transthoracic echocardiography is easier to perform and cheaper, but it also has limited availability for general population screening [10]. Currently, ECG is the most frequently used as a first screening tool to identify LVH [11]. The major limitation of ECG–LVH criteria, in the screening for LVH, is low sensitivity [12,13,14,15].

The aim of our study was to assess the importance of different approaches in the screening for LVH, including clinical data, chest radiograph parameters and ECG–LVH criteria used alone and in combination.

## 2. Materials and Methods

### 2.1. Study Population

We included patients with prevalent cardiovascular diseases who underwent CMR imaging in the Department of Diagnostic Imaging, University Hospital in Kraków (Poland) between 2011 and 2015 and had available chest radiograph taken in the posterior-anterior projection in the upright position during inspiration in medical documentation. Baseline patient clinical and demographic data and medication history were obtained from a structured medical records review. The study was approved by the local ethics committee.

### 2.2. CMR Imaging

CMR images were obtained using a 1.5 Tesla GE Signa HDxt scanner (General Electric, Milwaukee, WI, USA). The fast imaging employing steady-state acquisition (FIESTA) cine technique and other techniques such as those reported previously were implemented [16,17]. Left ventricular ejection fraction (LVEF), left ventricular end-systolic volume (LVESV), LVEDV and LVM were assessed with the use of standard volumetric techniques and calculated with commercially available QMass^®^ MR analysis software, version 7.6 (Medis Medical Imaging Systems bv, Leiden, The Netherlands) [16]. LVM was indexed for body surface area (BSA) and LVH diagnosed based on CMR (CMR–LVH) was defined as LVM/BSA >72 g/m^2^ in men or >55 g/m^2^ in women [18].

### 2.3. Chest Radiographs

Chest radiographs were taken in the posterior-anterior projection in the upright position during inspiration. The TCD was measured by drawing a vertical line through the vertebral bodies and calculating the sum of segments drawn perpendicular from the midline to the farthest edge of the cardiac silhouette in both directions [19,20]. CTR was calculated as a ratio of TCD to the greatest horizontal distance between the inner borders of the ribs within the chest [20,21]. Abnormal chest radiograph parameters, indicating cardiac enlargement, were determined as a CTR > 0.5 and as a TCD ≥ 155 mm in men or ≥145 mm in women [2,3,4].

### 2.4. ECG Analysis

ECG data were available for 38 patients. Standard 12-lead ECGs were recorded at 25 mm/s paper speed and calibration of 10 mm/mV and were assessed by an investigator who was primarily blinded to the patient’s CMR data. We evaluated 10 different ECG criteria for the LVH, as described previously [16]. In the current analysis, we used the following ECG–LVH criteria: R wave amplitude in aVL + S wave amplitude in V3 (Cornell voltage) >2.8 mV for men or >2.0 mV for female [22], (R wave amplitude in aVL + S wave amplitude in V3) × QRS duration for men or (R wave amplitude in aVL + S wave amplitude in V3 + 0.8 mV) × QRS duration for women (Cornell (voltage-duration) product) ≥244.0 mV × ms [23], S wave amplitude in V1 + R wave amplitude in V5 or V6 (Sokolow-Lyon voltage) >3.5 mV [24], the deepest S-wave in any single lead + S wave amplitude in V4 (Peguero-Lo Presti) ≥2.3 mV for female or ≥2.8 mV for men [13], and at least one positive ECG–LVH criterion (when all 10 previously described ECG criteria were applied together) [16].

### 2.5. A Fortified Method to Screen for Left Ventricular Hypertrophy

We assessed the rule described by Park et al., which included combining female sex, selected cardiovascular risk factors (age ≥65 years and body mass index (BMI) ≥25 kg/m^2^), chest radiograph (CTR ≥ 0.5) and the ECG–LVH criterion (Sokolow-Lyon voltage ≥3.5 mV) [5]. A score of ≥2 points indicated LVH [5].

### 2.6. Statistical Analysis

Continuous variables were tested with the t test or the Mann–Whitney test and were presented as means ± standard deviations (SD) or medians and interquartile ranges (IQR), when appropriate. Associations between categorical variables were assessed using the Pearson χ^2^ test or Fisher exact test, and results were given as numbers and percentages. Spearman rank correlation was used to measure the degree of association between two continuous variables. Receiver operating characteristics (ROC) analysis was used to find out the best variables to discriminate between patients with and without CMR–LVH. Based on the performed analyses, we modified the score proposed by Park et al. and proposed a modified score for LVH screening. Comparison of proportions was implemented to test for differences in positive LVH diagnoses. The different method used for screening for LVH was compared using the McNemar test. The agreement between the chest radiograph, ECG–LVH and combined criteria and the diagnosis of CMR–LVH was compared using the Cohen’s kappa coefficient. Additionally, specificity, sensitivity, positive predictive value, negative predictive value, accuracy and likelihood ratios were calculated for CTR, TCD, ECG–LVH criteria and combined criteria for LVH screening. Statistical significance was defined as *p* < 0.05 for all tests. Statistical analyses were carried out using IBM SPSS Statistics (version 25, IBM Corp., Armonk, NY, USA), Statistica (version 13.3, TIBCO Software Inc., Palo Alto, CA, USA) to compare areas under the curves (AUC) in analyses of receiver operating characteristics (Hanley and McNeil formula), while confidence intervals (CI) were calculated, and comparison of proportions were performed using MedCalc software (version 20.110, Medcalc Software Ltd., Ostend, Belgium) (available at: https://www.medcalc.org/, accessing date: 12 March 2022).

## 3. Results

### 3.1. Study Population

The study group consisted of 55 patients (16.4% female) with a median age of 42.0 (29.0–63.0) years. Among the studied patients, hypertension was present in 23 (41.8%), dyslipidemia in 21 (38.2%), diabetes in 8 (14.5%), atrial fibrillation in 8 (14.5%), chronic kidney disease in 6 (10.9%), coronary artery disease in 22 (40.0%) and heart failure in 35 (63.6%). Median LVEF was 42.7 (24.7–60.0)%, LVEDV was 189.6 (162.3–276.3) ml, LVESV was 94.3 (65.6–205.5) ml, while LVM was 161.0 (127.3–225.6) g. The prevalence of CMR–LVH was 72.7%. The threshold of age of ≥40 years, as a predictor of LVH, was determined based on ROC analysis (Youden index = 0.38; AUC, 0.679; 95% CI, 0.517–0.841; *p* = 0.04). Baseline characteristics of the patients with or without CMR–LVH, based on indexed LVM for BSA, is shown in Table 1.

### 3.2. Associations between Radiographic and Basic CMR Variables

**Figure 1:**
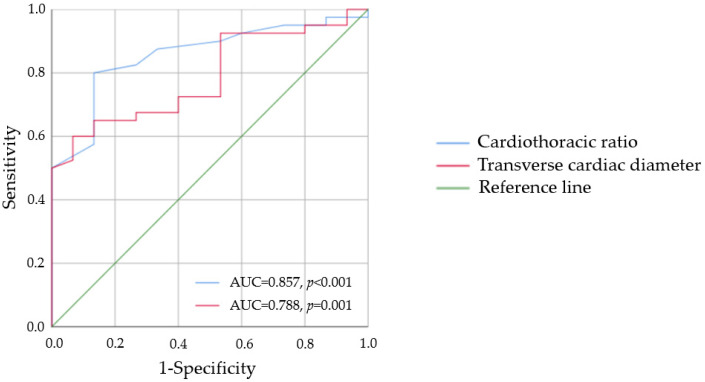
Area under the curve of chest radiograph criteria representing the predictive performance of left ventricular hypertrophy based on left ventricular mass indexed by body surface area. AUC—area under the curve.

Both CTR and TCD showed positive correlations with LVM (R = 0.53, *p* < 0.001; R = 0.73, *p* < 0.001, respectively). Similarly, positive correlations were observed between these radiographic parameters and LVEDV (R = 0.47, *p* < 0.001; R = 0.61, *p* < 0.001), and LVESV (R = 0.52, *p* < 0.001; R = 0.66, *p* < 0.001), while both CTR and TCD showed significant negative correlations with LVEF (R = −0.58, *p* < 0.001; R = −0.64, *p* < 0.001). Receiver operating characteristics analysis showed that both the CTR and TCD (CTR: area under curve: AUC = 0.857, *p* < 0.001; TCD: AUC = 0.788, *p* = 0.001; Figure 1) were predictors for CMR–LVH. When comparing AUC for these criteria, no significant difference between them was observed (*p* = 0.14).

### 3.3. Characteristics of the Patients with Normal and Increased Chest Radiograph Indicators of Cardiac Size Enlargement

Patients with CTR > 0.5 were older and had higher BMI (60.0 (40.5–69.5) vs. 36.0 (27.0–60.5) years, *p* = 0.009; 29.0 ± 5.6 vs. 24.8 ± 3.7 kg/m^2^, *p* = 0.002; Table 2) when compared to patients with normal CTR. In patients with CTR > 0.5, atrial fibrillation and heart failure were more common than in the remaining patients (35.3% vs. 5.3%, *p* = 0.008 and 94.1% vs. 50.0%, *p* = 0.002, respectively). Similarly, patients with TCD ≥ 155 mm in men or ≥ 145 mm in women were older when compared to patients with normal TCD (60.0 (37.0–70.0) vs. 32.5 (27.0–44.3) years, *p* < 0.001; Table 2). However, there was no significant difference in BMI between these two groups (27.0 (24.0–30.2) vs. 23.8 (22.1–27.5) kg/m^2^, *p* = 0.08). On the other hand, in patients with TCD ≥ 155 mm in men or ≥145 mm in women, cardiovascular disease risk factors and comorbidities (heart failure, hypertension and dyslipidemia) were observed more frequently when compared to the remaining patients (80.6% vs. 41.7%, *p* = 0.005; 54.8% vs. 25.0%, *p* = 0.03, 51.6% vs. 20.8%, *p* = 0.03, respectively).

### 3.4. Radiographic and Electrocardiographic Criteria in the Screening for LVH

When comparing a group of 38 patients with available ECG data, we observed that only the Peguero-Lo Presti criterion was more frequently positive in patients with CMR–LVH (56.0% vs. 15.4%, *p* = 0.02). Performed analyses (the McNemar test) have shown that diagnoses of cardiac enlargement made with TCD, but not CTR, were consistent with CMR–LVH. From ECG–LVH criteria only, at least one positive ECG criterion was consistent with CMR in LVH diagnosis (Table 3).

From chest radiograph parameters, the highest sensitivity was observed for TCD; however, specificity of this parameter was lower when compared to CTR (56.0 (34.9–75.6)% vs. 28.0 (12.1–49.4)%; 76.9 (46.2–95.0)% vs. 100.0 (75.3–100.0)%, respectively; Table 4). Moreover, the sensitivity for TCD was the same as sensitivities for the Peguero-Lo Presti criterion and at least one positive ECG-LVH criterion and higher than sensitivities for Cornell criteria and the Sokolow–Lyon voltage criterion (Table 4). The positive predictive value, negative predictive value, accuracy, positive and negative likelihood ratio of all the analyzed chest radiographs and ECG–LVH criteria are shown in Table 4.

### 3.5. A Novel Screening Tool for LVH

**Figure 2:**
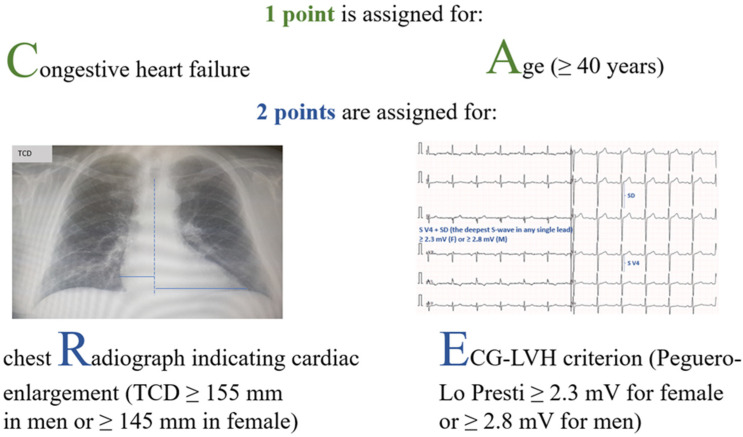
The Congestive heart failure, Age, chest Radiograph and Electrocardiographic data (CAR_2_E_2_) score assessment.

We modified the Park rule based on the results of our statistical analyses. We replaced CTR by TCD and the Sokolow–Lyon voltage criterion by other ECG–LVH criteria (at least one ECG–LVH criterion positive and Peguero-Lo Presti criterion; Model 1 and 2, respectively). Moreover, we tested whether our new score based on a point system in which 1 point is assigned for heart failure and age ≥40 years, and 2 points are assigned for chest radiograph indicating cardiac enlargement (TCD indicating cardiac enlargement) and positive Peguero-Lo Presti criterion (CAR_2_E_2_ score), improves prediction of LVH compared to other approaches used for LVH screening. The methodology of the CAR_2_E_2_ score calculation is depicted in Figure 2.

### 3.6. Combined Criteria in the Screening for LVH

When comparing a group of 38 patients with the available ECG data, we observed that only Model 2 at the score ≥2 and CAR_2_E_2_ score of ≥3 were more frequently positive in patients with CMR–LVH (60.0% vs. 23.1%, *p* = 0.03; 72.0% vs. 23.1%, 0.004, respectively). When comparing these 2 differentiating criteria using a comparison of proportion test, there was no difference between them. Performed analyses (the McNemar test) demonstrated that Model 1 at the score ≥2, Model 2 at the score ≥2 and CAR_2_E_2_ score at the score ≥3 were in agreement with CMR in LVH diagnosis (Table 5). However, the strongest agreement was found for the CAR_2_E_2_ score, when compared to TCD, with at least one positive ECG-LVH criterion, Model 1 at a score of ≥2 and Model 2 at a score of ≥2 (κ coefficient of 0.46 vs. 0.29; 0.29; −0.12; −0.12, respectively).

The rule described by Parke et al. combining the risk factors, ECG and chest radiograph criteria showed a sensitivity of 40.0 (21.1–61.3)% and specificity of 84.6 (54.6–98.1)%. After modification of this rule by replacing CTR by TCD and replacing the Sokolow–Lyon voltage by other ECG criterion (at least one ECG-LVH criterion positive and Peguero-Lo Presti criterion), we observed a sensitivity of 60.0 (38.7–78.9)% in both of cases (Table 6). The best sensitivity was observed for a CAR_2_E_2_ score of ≥3 (72.0 (50.6–87.9)%). The positive predictive value, negative predictive value, accuracy and positive and negative likelihood ratios of all the analyzed combined criteria are shown in Table 6.

**Figure 3:**
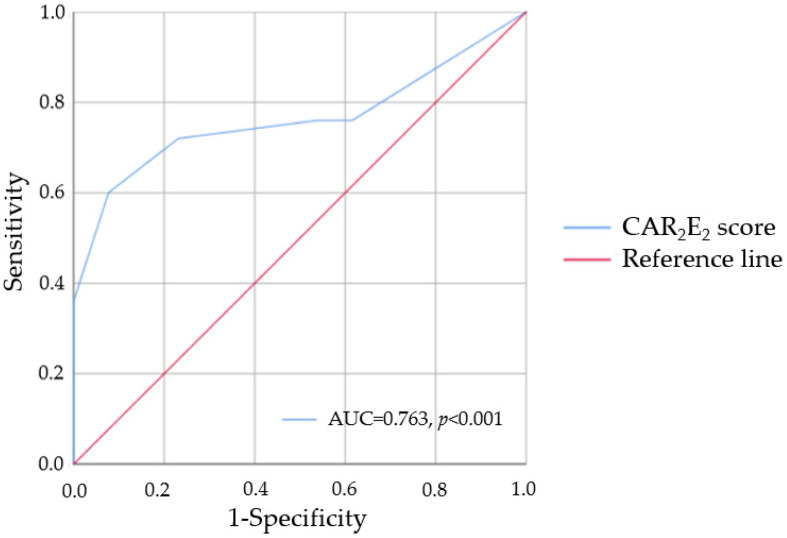
Area under the curve of CAR_2_E_2_ score representing the predictive performance of left ventricular hypertrophy based on left ventricular mass indexed by body surface area. AUC—area under the curve.

Receiver operating characteristics analysis showed that only a CAR_2_E_2_ score of ≥3 (AUC = 0.763, *p* < 0.001; Figure 3) was a predictor of CMR–LVH. Additionally, in ROC analysis, a CAR_2_E_2_ score of <3 points was a predictor of a lack of CMR–LVH (AUC = 0.745, *p* = 0.005).

Importantly, when we tested the CAR_2_E_2_ score, and used CTR in the place of TCD (as “R_2_“) and all investigated ECG–LVH criteria in place of Peguero-Lo Presti criterion (as “E_2_“) interchangeably, there was no observed difference regarding AUC in ROC analysis in the prediction of CMR–LVH.

## 4. Discussion

There is increasing interest in the use of markers and risk scores as well as in testing their clinical applicability in cardiovascular medicine [25,26,27,28,29,30,31,32,33]. Several factors have been found to be associated with LVH assessed by different modalities [7,16]. Studies performed so far have demonstrated that there is a relationship between CTR and LVM measured by echocardiography [5,34,35]. Rayner et al. revealed that CTR (R = 0.34, *p* < 0.02) was independently correlated with LVM when evaluated with this modality [34]. Similar correlation of CTR and LVM determined by echocardiography (R = 0.43, *p* < 0.01) was found in another study performed by Buba et al. [35]. However, research investigating the association between CTR and LVM determined by CMR is lacking. Our study demonstrates that both CTR and TCD were predictors of CMR–LVH in receiver operating characteristics analyses and have shown moderate correlation not only with the LVM, but also with LVEDV, LVESV and LVEF. However, the strongest correlations were observed for TCD. Similarly to our results, Morales et al. have shown that while a good relation was found between LVEDV and TCD, this relation was less significant for the CTR [1]. These data suggest that TCD may be a better indicator than CTR of LV dimension and hypertrophy. However, it should also be remembered that CTR and TCD do not take into account changes in the size of the heart throughout the cardiac cycle.

Moreover, CTR and TCD may be influenced by several other factors, such as pericardial fat, elevation of the diaphragm either due to poor inspiration or obesity, the breathing phase or thoracic alterations (e.g., severe scoliosis, pectus excavatum) [2,36,37,38,39]. However, TCD seems to be influenced by deformities of the chest (especially affecting the transverse chest diameter) to a lesser extent than CTR. Importantly, identifying factors explaining the discrepancies between chest radiograph parameters and CMR–LVH may improve diagnostic abilities. For example, right ventricular enlargement may cause greater TCD and CTR measures [40,41,42]. In a study of heart failure patients, Fukuta et al. revealed that increased CTR correlated more strongly with the size of the right ventricle than LV [41]. Recently, it has also been demonstrated that CTR could play a role in predicting right ventricular enlargement in patients with suspected pulmonary embolism during COVID-19 [42]. Elevation of the diaphragm due to high BMI can place the heart in a more horizontal position leading to an increase of TCD and CTR. Our study showed that patients with and without CMR-LVH did not significantly differ in BMI. Inversely, these parameters were significantly different when compared to patients with normal and higher CTR and trended toward higher BMI values in patients with increased TCD than patients with normal TCD. This suggests that especially high BMI may be responsible for some discrepancies between chest radiograph parameters and CMR–LVH. However, verification of this hypothesis requires further investigation.

Ribeiro et al., in a study on hypertensive patients, demonstrated that CTR had a sensitivity of 16.7% and a specificity of 88.3% for identifying the LVH [43]. For comparison, for ECG criteria (Romhilt–Estes point score system) sensitivity and specificity for the detection of LVH were 12.5% and 92.2%, respectively [43]. These findings show that chest radiographs may be useful for detecting LVH in hypertensive patients. Moreover, some studies suggest that the standard CRT criterion is not a good enough indicator of cardiac enlargement and therefore consider the introduction of another cut-off point for CTR or indicate TCD assessment as a better method [44,45,46]. Our study performed in patients with prevalent cardiovascular diseases revealed that from chest radiograph parameters, the highest sensitivity was observed for TCD, and this sensitivity was the same as for the Peguero-Lo Presti criterion and at least one positive ECG-LVH criterion. Moreover, the sensitivity of TCD was higher than sensitivities for Cornel criteria and the Sokolow–Lyon voltage criterion. These data suggest that chest radiographs may be valuable in screening for LVH not only in hypertensive patients but also in subgroups with prevalent cardiovascular diseases.

Increased LVM may impair LV function and might predict HF in some individuals [47,48]. Studies have shown that LVH is the most frequent myocardial abnormality associated with HF with preserved EF, and its prevalence increases with age [48,49]. Interestingly, we have shown that the combination of cardiovascular risk factors (heart failure and age ≥40 years) with chest radiograph parameters and ECG criteria may improve screening for LVH. After modification of a model proposed by Park et al. [5] by replacing the CTR by TCD and replacing the Sokolow–Lyon voltage by another ECG criterion (at least one ECG-LVH criterion positive and Peguero-Lo Presti criterion), we observed better sensitivity than the sensitivities of ECG and chest radiograph criteria solely. However, it should be mentioned that the fortified method for screening for LVH proposed by Park et al. was previously tested only in a hypertensive Asian population [5]. Thus, further studies on this topic performed on a larger and more diversified cohort are required. Moreover, we revealed that the novel score system that we have proposed, the CAR_2_E_2_ score, had the best sensitivity and may be considered as a fortified method for screening for LVH in daily clinical practice. A screening tool with high sensitivity may be preferred in the context of LVH (due to, e.g., very low/no risk, low cost and high availability of transthoracic echocardiography in many clinical settings) [50]. Due to there being no difference in AUC in ROC analysis, in the prediction of CMR–LVH, when CTR was used instead of TCD and all investigated ECG–LVH criteria instead of the Peguero-Lo Presti criterion, in clinical practice both of these radiological criteria and also investigated ECG–LVH criteria, besides the Peguero-Lo Presti criterion, might be useful and potentially used interchangeably. Our study also revealed that the CAR_2_E_2_ score may be useful for patients with fewer than 3 points by being a predictor of a lack of CMR–LVH. However, it is important to remember that these patients with a positive ECG–LVH criterion and/or chest radiograph suggestive of cardiac enlargement should not be excluded from further diagnostic processes. Despite the strongest agreement with CMR–LVH for a CAR_2_E_2_ score of ≥3, when compared to chest radiograph parameters indicating cardiac enlargement or ECG–LVH criteria, studies have shown that ECG and chest radiograph used alone are also useful in screening for LVH [16,36].

Identifying the underlying etiology of LVH is a common, and sometimes challenging clinical problem. It is especially crucial to differentiate physiological changes in the heart, e.g., of an athlete, with pathological forms of LVH, for example, in patients with aortic stenosis or some cardiomyopathies, and examinations included in the CAR_2_E_2_ score might provide clinical clues. Evaluation consisting of a detailed clinical history (e.g., older age in aortic stenosis patients), chest radiograph (e.g., aortic valve calcification and widening of the ascending aorta in aortic stenosis) and ECG (e.g., physiological in black/African athletes T wave inversions in V1-V4 accompanied by convex ST segment elevation) might be useful in clinical differentiation [37,51,52,53]. In echocardiography, we usually observe normal contractility, as well as normal global longitudinal strain, along with usually normal LV wall thickness in athletes [54], while specific alterations are observed in patients with aortic stenosis. Additionally, CMR, especially with late gadolinium enhancement, may provide further diagnostic clues and prognostic information [54,55,56].

There are several limitations of our study which should be mentioned. Chest radiograph, ECG and CMR studies were not always performed on the same day. Our study has a retrospective nature and relatively small group of included patients with cardiovascular diseases. Therefore, our results may not be representative for the whole population and should be confirmed in a larger group of patients. However, even in this relatively small group, we could find differences in the tested chest radiograph parameters, ECG–LVH criteria and combined approaches for LVH detection. Finally, we did not investigate associations of the CAR_2_E_2_ score with major adverse cardiovascular events. This should be evaluated in further studies. However, the components of our new score have proven the relationship with unfavorable clinical outcomes [29,33,36,57,58]. Therefore, CAR_2_E_2_ score is most likely related to increased clinical risk.

## 5. Conclusions

CAR_2_E_2_ score may improve prediction of LVH compared to other approaches, including chest radiograph parameters used on their own and in combination with selected clinical data and ECG–LVH criteria. Therefore, it may be useful in the screening for LVH in everyday clinical practice in patients with prevalent cardiovascular diseases.

## Figures and Tables

**Table 1:** Characteristics of the patients with and without left ventricular hypertrophy based on left ventricular mass indexed by body surface area.

Parameters	LVH; *n* = 40	No LVH; *n* = 15	*p* Value
Demographic characteristicsand anthropometric data			
Age (years)	51.5 (32.3–64.0)	33.0 (25.0–47.0)	0.04
Age ≥40 years	26 (65.0%)	4 (26.7%)	0.01
Age ≥65 years	11 (27.5%)	2 (13.3%)	0.48 *
Female sex, *n* (%)	7 (17.5%)	2 (13.3%)	1 *
BMI (kg/m^2^)	25.1 (22.1–29.0)	26.8 (23.1–28.7)	0.63
BMI ≥ 25 kg/m^2^, *n* (%)	20 (50.0%)	9 (60.0%)	0.5
BMI ≥ 30 kg/m^2^, *n* (%)	7 (17.5%)	3 (20.0%)	1 *
Cardiovascular diseases, comorbidities and risk factors, *n* (%)			
Heart failure	30 (75.0%)	5 (33.3%)	0.004
CAD	18 (45.0%)	4 (26.7%)	0.22
Myocardial infarction	10 (25.0%)	2 (13.3%)	0.35
Diabetes mellitus	7 (17.5%)	1 (6.7%)	0.42 *
Hypertension	18 (45.0%)	5 (33.3%)	0.44
Dyslipidemia	17 (42.5%)	4 (26.7%)	0.28
Smoking	11 (27.5%)	2 (13.3%)	0.48
Atrial fibrillation	7 (17.5%)	1 (6.7%)	0.42 *
CKD	5 (12.5%)	1 (6.7%)	1 *
CMR parameters			
LVEF (%)	29.7 (23.4–50.5)	59.5 (50.9–66.2)	<0.001
LVM (g)	173.0 (152.9–234.0)	126.0 (114.4–134.5)	<0.001
LVEDV (mL)	228.0 (169.4–322.4)	169.5 (123.0–189.6)	0.002
LVEDV/BSA > 117 (M), >101 (F), *n* (%)	20 (50.0%)	0 (0.0%)	0.001
LVESV (mL)	124.6 (84.7–261.7)	65.6 (58.3–74.1)	<0.001
Chest radiograph data			
CTR	0.50 ± 0.07	0.42 ± 0.04	<0.001
CTR > 0.5, *n* (%)	17 (42.5%)	0 (0.0%)	0.002 *
TCD (mm)	167.7 ± 26.9	141.4 ± 16.9	0.001
TCD ≥ 155 mm (M), ≥145 mm (F), *n* (%)	28 (70.0%)	3 (20.0%)	0.001

Data are presented as mean ± standard deviation or median (interquartile range) or number (percentage). * Fisher exact test (exact significance, 2-sided). BMI—body mass index; BSA—body surface area; CAD—coronary artery disease; CKD—chronic kidney disease; CMR—cardiac magnetic resonance; CTR—cardiothoracic ratio; F—female; LVEDV—left ventricular end-diastolic volume; LVEF—left ventricular ejection fraction; LVESV—left ventricular end-systolic volume; LVM—left ventricular mass; M—male; TCD—transverse cardiac diameter.

**Table 2:** Baseline characteristics of the patients with normal and increased chest radiograph indicators of cardiac size enlargement.

Parameters	CTR > 0.5 (*n* = 17)	CTR ≤ 0.5 (*n* = 38)	*p* Value	TCD ≥ 155 mm (M) or ≥ 145 mm (F) (*n* = 31)	TCD < 155 mm (M) or < 145 mm (F) (*n* = 24)	*p* Value
Demographic characteristicsand anthropometric data						
Age (years)	60.0 (40.5–69.5)	36.0 (27.0–60.5)	0.009	60.0 (37.0–70.0)	32.5 (27.0–44.3)	<0.001
Age ≥40 years	14 (82.4%)	16 (42.1%)	0.006	23 (74.2%)	7 (29.2%)	0.001
Age ≥65 years	6 (35.3%)	7 (18.4%)	0.19 *	12 (38.7%)	1 (4.2%)	0.003
Female sex, *n* (%)	2 (11.8%)	7 (18.4%)	0.71 *	3 (9.7%)	6 (25.0%)	0.16 *
BMI (kg/m^2^)	29.0 ± 5.6	24.8 ± 3.7	0.002	27.0 (24.0–30.2)	23.8 (22.1–27.5)	0.08
BMI ≥ 25 kg/m^2^, *n* (%)	14 (82.4%)	15 (39.5%)	0.003	20 (64.5%)	9 (37.5%)	0.047
BMI ≥ 30 kg/m^2^, *n* (%)	6 (35.3%)	4 (10.5%)	0.05 *	7 (22.6%)	3 (12.5%)	0.49 *
Cardiovascular diseases, comorbidities and risk factors, *n* (%)						
Heart failure	16 (94.1%)	19 (50.0%)	0.002	25 (80.6%)	10 (41.7%)	0.005
CAD	10 (58.8%)	12 (31.6%)	0.06	16 (51.6%)	6 (25.0%)	0.06
Myocardial infarction	4 (23.5%)	8 (21.1%)	1 *	9 (29.0%)	3 (12.5%)	0.19
Diabetes mellitus	5 (29.4%)	3 (7.9%)	0.09 *	6 (19.4%)	2 (8.3%)	0.44 *
Hypertension	8 (47.1%)	15 (39.5%)	0.6	17 (54.8%)	6 (25.0%)	0.03
Dyslipidemia	9 (52.9%)	12 (31.6%)	0.13	16 (51.6%)	5 (20.8%)	0.03
Smoking	3 (17.6%)	10 (26.3%)	0.73 *	7 (22.6%)	6 (25.0%)	1
Atrial fibrillation	6 (35.3%)	2 (5.3%)	0.008 *	6 (19.4%)	2 (8.3%)	0.44 *
CKD	2 (11.8%)	4 (10.5%)	1 *	5 (16.1%)	1 (4.2%)	0.22 *
CMR parameters						
LVEF (%)	25.6 ± 9.1	49.2 ± 16.7	<0.001	29.5 (21.0–46.7)	58.2 (42.7–63.0)	<0.001
LVM (g)	233.6 (181.9–271.3)	134.7 (120.9–167.0)	<0.001	201.6 ± 62.8	136.8 ± 29.1	<0.001
LVM/BSA > 72 g/m^2^ (M) or >55 g/m^2^ (F), *n* (%)	17 (100.0%)	23 (60.5%)	0.002 *	28 (90.3%)	12 (50.0%)	0.001
LVEDV (mL)	317.3 (239.7–392.0)	175.2 (140.2–199.5)	<0.001	228.8 (168.7–355.6)	173.9 (136.3–194.6)	0.005
LVEDV/BSA > 117 (M), >101 (F), *n* (%)	14 (82.4%)	6 (15.8%)	<0.001	17 (54.8%)	3 (12.5%)	0.002
LVESV (mL)	239.4 (152.4–313.1)	82.4 (63.3–112.4)	<0.001	173.9 (82.1–271.3)	71.7 (58.0–104.9)	0.001
Chest radiograph data						
CTR	0.57 ± 0.05	0.44 ± 0.04	<0.001	0.53 ± 0.06	0.42 ± 0.04	<0.001
TCD (mm)	192.3 ± 17.9	146.3 ± 16.3	<0.001	171.0 (161.8–194.3)	140.7 (130.3–149.5)	<0.001
TCD ≥ 155 mm (M), ≥145 mm (F), *n* (%)	17 (100%)	14 (36.8%)	<0.001	17 (54.8%)	0 (0.0%)	<0.001

Data are presented as mean ± standard deviation or median (interquartile range) or number (percentage). * Fisher exact test (exact significance, 2-sided). BMI—body mass index; BSA—body surface area; CAD—coronary artery disease; CKD—chronic kidney disease; CMR—cardiac magnetic resonance; CTR—cardiothoracic ratio; F—female; LVEDV—left ventricular end-diastolic volume; LVEF—left ventricular ejection fraction; LVESV—left ventricular end-systolic volume; LVM—left ventricular mass; M—male; TCD—transverse cardiac diameter.

**Table 3:** Radiographic and electrocardiographic criteria in the screening for left ventricular hypertrophy in patients with and without left ventricular hypertrophy based on left ventricular mass indexed by body surface area.

Parameters	LVH; *n* = 25		No LVH; *n* = 13		McNemar Test
	TP	FN	FP	TN	
Chest radiograph indicators of cardiac size enlargement					
CTR > 0.5	7 (28.0%)	18 (72.0%)	0 (0.0%)	13 (100.0%)	<0.001
TCD ≥ 155 mm (M), ≥145 mm (F)	14 (56.0%)	11 (44.0%)	3 (23.1%)	76.9%)	0.06
ECG-LVH criteria					
Cornell voltage	1 (4.0%)	24 (96.0%)	0 (0.0%)	13 (100.0%)	<0.001
Cornell product	2 (8.0%)	23 (92.0%)	0 (0.0%)	13 (100.0%)	<0.001
Peguero-Lo Presti criterion	14 (56.0%)	11 (44.0%)	2 (15.4%)	11 (84.6%)	0.02
Sokolow-Lyon voltage	3 (12.0%)	22 (88.0%)	1 (7.7%)	12 (92.3%)	<0.001
At least one positive ECG-LVH criterion	14 (56.0%)	11 (44.0%)	3 (23.1%)	10 (76.9%)	0.06

Data are presented as number (percentage). CTR—cardiothoracic ratio; ECG—electrocardiographic; FN—false negative; FP—false positive; LVH—left ventricular hypertrophy; TCD—transverse cardiac diameter; TN—true negative; TP—true positive. Calculations were made for 38 patients.

**Table 4:** Radiographic and electrocardiographic criteria in the screening for left ventricular hypertrophy and their sensitivity, specificity, positive predictive value, negative predictive value, accuracy, positive likelihood ratio and negative likelihood ratio. Data are shown for indexed left ventricular mass by body surface area.

Parameters	Sensitivity (%) (95% CI)	Specificity (%) (95% CI)	PPV (%) (95% CI)	NPV (%) (95% CI)	Accuracy (%) (95% CI)	PLR (95% CI)	NLR (95% CI)
Chest radiograph indicators of cardiac size enlargement							
CTR > 0.5	28.0 (12.1–49.4)	100.0 (75.3–100.0)	100.0 *	41.9 (36.1–48.0)	52.6 (35.8–69.0)	*	0.7 (0.6–0.9)
TCD ≥ 155 mm (M), ≥145 mm (F)	56.0 (34.9–75.6)	76.9 (46.2–95.0)	82.4 (62.0–93.0)	47.6 (34.8–60.8)	63.2 (46.0–78.2)	2.4 (0.9–7.0)	0.6 (0.3–1.0)
ECG-LVH criteria							
Cornell voltage	4.0 (0.1–20.4)	100.0 (75.3–100.0)	100.0 *	35.1 (33.3–37.0)	36.8 (21.8–54.0)	*	1.0 (0.9–1.0)
Cornell product	8.0 (1.0–26.0)	100.0 (75.3–100.0)	100.0 *	36.1 (33.5–38.8)	39.5 (24.0–56.6)	*	0.9 (0.8–1.0)
Peguero-Lo Presti criterion	56.0 (34.9–75.6)	84.6 (54.6–98.1)	87.5 (65.1–96.3)	50.0 (37.8–62.2)	65.8 (48.7–80.4)	3.6 (1.0–13.6)	0.5 (0.3–0.9)
Sokolow-Lyon voltage	12.0 (2.6–31.2)	92.3 (64.0–99.8)	75.0 (25.7–96.3)	35.3 (30.6–40.3)	39.5 (24.0–56.6)	1.6 (0.2–13.6)	1.0 (0.8–1.2)
At least one positive ECG-LVH criterion	56.0 (34.9–75.6)	76.9 (46.2–95.0)	82.4 (62.0–93.0)	47.6 (34.8–60.8)	63.2 (46.0–78.2)	2.4 (0.9–7.0)	0.6 (0.3–1.0)

Data are presented as percentage (95% CI) or number (95% CI). CI—confidence interval; CTR—cardiothoracic ratio; ECG—electrocardiographic; LVH—left ventricular hypertrophy; NLR—negative likelihood ratio; NPV—negative predictive value; PLR- positive likelihood ratio; PPV—positive predictive value; TCD—transverse cardiac diameter. Calculations were made for 38 patients. *—95% CI, PLR and/or NLR not available.

**Table 5:** Combined criteria in the screening of left ventricular hypertrophy in patients with and without left ventricular hypertrophy based on left ventricular mass indexed by body surface area.

Parameters	LVH; *n* = 25		No LVH; *n* = 13		McNemar Test
	TP	FN	FP	TN	
Combined Rules with Use the Clinical Risk Factors, Chest Radiograph and ECG-LVH Criteria					
Model proposed by Park et al. [5] at score ≥2 ^†^	10 (40.0%)	15 (60.0%)	2 (15.4%)	11 (84.6%)	0.002
Model 1 at score ≥2 ^‡^	15 (60.0%)	10 (40.0%)	4 (30.8%)	9 (69.2%)	0.18
Model 2 at score ≥2 ^#^	15 (60.0%)	10 (40.0%)	3 (23.1%)	10 (76.9%)	0.09
CAR_2_E_2_ score ≥3 ^##^	18 (72.0%)	7 (28.0%)	3 (23.1%)	10 (76.9%)	0.344

Data are presented as number (percentage). ^†^ Includes female sex, age ≥65 years, BMI ≥ 25 kg/m^2^, Sokolow-Lyon voltage ≥3.5 mV, and CTR ≥ 0.5. ^‡^ Includes female sex, age ≥65 years, BMI ≥ 25 kg/m^2^, at least one ECG-LVH criterion positive, and TCD ≥ 155 mm (M), ≥145 mm (F). ^#^ Includes female sex, age ≥65 years, BMI ≥ 25 kg/m^2^, Peguero-Lo Presti criterion ≥2.3 mV (F) or ≥2.8 mV (M), and TCD ≥ 155 mm (M), ≥145 mm (F). ^##^ Includes congestive heart failure, age (≥40 years), Peguero-Lo Presti criterion: the deepest S-wave in any single lead + S wave amplitude in V4 ≥ 2.3 mV (F) or ≥2.8 mV (M), and TCD ≥ 155 mm (M), ≥145 mm (F). ECG—electrocardiographic; FN—false negative; FP—false positive; LVH—left ventricular hypertrophy; TN—true negative; TP—true positive. Calculations were made for 38 patients.

**Table 6:** Combined criteria for screening of left ventricular hypertrophy and their sensitivity, specificity, positive predictive value, negative predictive value, accuracy, positive likelihood ratio and negative likelihood ratio. Data are shown for indexed left ventricular mass by body surface area.

**Parameters**	**Sensitivity (%)** **(95% CI)**	**Specificity (%)** **(95% CI)**	**PPV (%)** **(95% CI)**	**NPV (%)** **(95% CI)**	**Accuracy (%)** **(95% CI)**	**PLR** **(95% CI)**	**NLR** **(95% CI)**
Combined Rules with Use the Clinical Risk Factors, Chest Radiograph and ECG-LVH Criteria							
Model proposed by Park et al. [5] score ≥2 ^†^	40.0 (21.1–61.3)	84.6 (54.6–98.1)	83.3 (56.2–95.1)	42.3 (33.1–52.1)	55.3 (38.3–71.4)	2.6 (0.7–10.2)	0.7 (0.5–1.1)
Model 1 score ≥2 ^‡^	60.0 (38.7–78.9)	69.2 (38.6–90.9)	79.0 (61.0–90.0)	47.4 (33.0–62.2)	63.2 (46.0–78.2)	2.0 (0.8–4.7)	0.6 (0.3–1.1)
Model 2 score ≥2 ^#^	60.0 (38.7–78.9)	76.9 (46.2–95.0)	83.3 (63.8–93.4)	50.0 (36.2–63.8)	65.8 (48.7–80.4)	2.6 (0.9–77.4)	0.5 (0.3–0.9)
CAR_2_E_2_ score ≥3 ^##^	72.0 (50.6–87.9)	76.9 (46.2–95.0)	85.7 (68.3–94.3)	58.8 (41.6–74.1)	73.7 (57.0–86.6)	3.1 (1.1–8.7)	0.4 (0.2–0.7)

Data are presented as percentage (95% CI) or number (95% CI). ^†^ Includes female sex, age ≥65 years, BMI ≥ 25 kg/m^2^, Sokolow-Lyon voltage criterion: S wave amplitude in V1 + R wave amplitude in V5 or V6 ≥ 3.5 mV, and CTR ≥ 0.5. ^‡^ Includes female sex, age ≥65 years, BMI ≥ 25 kg/m^2^, at least one ECG-LVH criterion positive, and TCD ≥ 155 mm (M), ≥145 mm (F). ^#^ Includes female sex, age ≥65 years, BMI ≥ 25 kg/m^2^, Peguero-Lo Presti criterion: the deepest S-wave in any single lead + S wave amplitude in V4 ≥ 2.3 mV (F) or ≥2.8 mV (M), and TCD ≥ 155 mm (M), ≥145 mm (F). ^##^ Includes congestive heart failure, age ≥ 40 years, Peguero-Lo Presti criterion, and TCD ≥ 155 mm (M), ≥145 mm (F). CI—confidence interval; ECG—electrocardiographic; LVH—left ventricular hypertrophy; NLR—negative likelihood ratio; NPV—negative predictive value; PLR- positive likelihood ratio; PPV—positive predictive value. Calculations were made for 38 patients.

## Data Availability

Not applicable.
